# Twisted loop mattress suture

**DOI:** 10.1308/003588412X13171221591259d

**Published:** 2012-05

**Authors:** LS Rees, B Sommerlad

**Affiliations:** Great Ormond Street HospitalLondon, UK

## BACKGROUND

**Figure 1:**
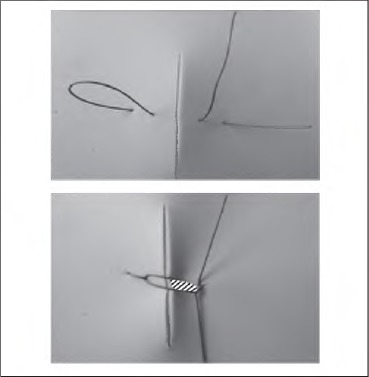
Standard loop mattress suture. Zone of strangulation shown hatched.

The advantages of the loop mattress suture have been described.^1^,^2^ It is commonly used for combined closure of skin and subcutaneous tissue and has been very useful in palate repair.^3^ We have, however, on occasion noted a small area of necrosis within the loop at the edges of the wound (Fig 1). This is more commonly seen with ‘springy’ monofilament sutures, particularly those of heavier gauge. We describe a simple modification to address this problem.

## TECHNIQUE

**Figure 2:**
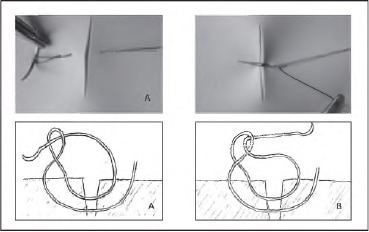
Twisted loop mattress suture shown from above demonstrating the elimination of the zone of strangulation and diagrammatically in cross section. Note the direction the needle passes through the loop depends on the direction in which the loop has been twisted. If the loop is twisted in a clockwise direction, the needle passes from back to front through the loop (A). If the loop is twisted anticlockwise, the needle passes front to back (B).

When placing a loop mattress suture, twisting the loop through 180º minimises the area of potential strangulation without loss of the other advantages of this suture. The needle is passed through the wound four times as in the traditional vertical mattress suture. The emergent thread is taken back over the suture line, the loop twisted through 180º and the needle passed through the loop from the direction to which the loop has been twisted. For example, if the loop is on the left side of the wound and twisted clockwise, the needle is passed from back to front through the loop and, if twisted anticlockwise, the needle is passed from front to back (Fig 2).

## DISCUSSION

We would advocate this modification of the loop mattress suture in reducing potential strangulation. Removal of the stitch requires precise division of the suture opposite the knot and is therefore perhaps best applied when an absorbable suture is being used. We have found it particularly useful in cleft palate repair.

## References

[1] Gault DT, Brain A, Sommerlad BC, Ferguson DJ., Loop mattress suture BrJ Surg 1987 74 820 821 3311284 10.1002/bjs.1800740922

[2] Sommerlad BC, Gault D., Space-obliterating skin suture Plast Reconstr Surg 1999 103 1,101 10077135

[3] Sommerlad BC, A technique for cleft palate repair Plast Reconstr Surg 2003 112 1,542 1,548 14578783 10.1097/01.PRS.0000085599.84458.D2

